# Predictors of Caregiver Burden Among Carers of Suicide Attempt Survivors

**DOI:** 10.1027/0227-5910/a000836

**Published:** 2021-12-17

**Authors:** Myfanwy Maple, Sarah Wayland, Rebecca L. Sanford, Navjot Bhullar

**Affiliations:** ^1^ School of Health, Faculty of Medicine and Health, University of New England, Armidale, NSW, Australia; ^2^ School of Social Work and Human Service, Thompson Rivers University, Kamloops, BC, Canada; ^3^ School of Psychology, Faculty of Medicine and Health, University of New England, NSW, Armidale, Australia

**Keywords:** suicide, suicide attempt, carer, carer burden

## Abstract

**Abstract.** *Background:* Family members often provide informal care following a suicide attempt. Carers may be vulnerable to caregiver burden. Yet, little is known about what contributes to this. *Aims:* To determine the predictors of caregiver burden in those carers who support people who have attempted suicide. *Method:* An online survey of 435 participants assessed exposure to suicide, caring behaviors, and psychological variables and caregiver burden. *Results:* A multivariate model explained 52% of variance in caregiver burden. Being female, closeness to the person, impact of suicide attempt, frequency of contact pre-attempt, and psychological distress were positively associated with caregiver burden. Confidence in supporting the person after suicide attempt, perceived adequacy of healthcare the person received and the support the carer received, and suicidal ideation of the carer were negatively associated with caregiver burden. Moderation analysis suggested that carers with high levels of distress reported negative association between suicidal ideation and caregiver burden. *Limitations:* The cross-sectional online survey design of self-identified carers is a limitation of the study. *Conclusion:* Carers are highly distressed, and if unsupported report increased suicide ideation. In their caring roles they may have contact with support services, thus attending to their needs may ameliorate caregiver burden and associated negative outcomes.

Suicide remains a significant public health issue in Australia with over 3,300 people dying in 2019 (Australian Bureau of Statistics [ABS], 2020). The causes of suicide are multifactorial, and for every suicide death, it is estimated there are more than 20 attempts (World Health Organization, 2014). While individuals may require medical care following a suicide attempt, it is family members or close friends (hereafter “carers”) who often provide informal care and support following a suicide attempt (Hom et al., 2015; Van Orden et al., 2010). Carers provide an important protective factor in reducing the risk of further suicide attempts and help facilitate recovery (Pereira et al., 2018). However, in providing support to those who have attempted suicide (hereafter “supported person”), carers are vulnerable to adverse physical and psychological outcomes such as burnout, fatigue, trauma, and reduced health status, collectively described as “caregiver burden” (Buus et al., 2014). This vulnerability can also extend to suicide risk (Maple et al., 2019). Since the main emphasis of care tends to be on the suicidal person, carers may feel isolated as their concerns are largely hidden (McLaughlin et al., 2014). The factors known to ameliorate or exacerbate the demands on carers include their relationship, contact (Buus et al., 2014), and the opportunity to develop a closer relationship with the supported person (Sarris et al., 2020). However, a close relationship between carer and supported person can increase pressure on carers to consistently monitor for suicide risk (Owens et al., 2011). These activities require carers to approach the role from multiple perspectives, which acknowledge the competing interests they must attend to (Wayland et al., 2020).

Receiving timely, adequate professional support can help reduce caregiver burden. However, carers may not feel comfortable or able to disclose suicidal behaviors of the supported person due to fear of stigma and shame (McLaughlin et al., 2016; Spillane, 2019). Carers have reported exposure to stigmatizing views from healthcare staff when seeking help, with some staff not taking their concerns seriously (Cerel et al., 2006) impeding carers' ability to seek help (Castelli Dransart & Guerry, 2017). Further, healthcare staff may be mandated to protect client confidentiality preventing them from disclosing information about the person the carer is supporting, which can be unhelpful for carers left to provide support following discharge from clinical care (McLaughlin et al., 2014). These tensions can impede clear communication between staff and carers, resulting in suboptimal care. This can be exacerbated where perceived failure to receive sufficient support from professionals exists (Wayland et al., 2020). Adding further complexity for carers are other commitments they are often balancing, including paid work and other family commitments, which can result in negative physical and mental health outcomes (Kenny et al., 2014) and caregiver burnout.

There are currently few evidence-based interventions to address and reduce caregiver burden (Perlick et al., 2016). Identifying those at risk of caregiver burnout or distress is required to provide a strong foundation upon which future supportive interventions can be developed. Thus, this study aimed to identify predictors of caregiver burden experienced as a result of caring. Given the literature indicating the complexity of providing care to someone after a suicide attempt, we hypothesized that carers would experience high caregiver burden if they also report closer relationship, more frequent contact, greater impact of the suicide, inadequate healthcare support for the supported person and support they received, increased psychological distress and suicidal thoughts. We further hypothesized that those who hold more stigmatizing views of suicide, who were less confident talking to the person and others about the attempt, and who supported the person after the attempt would experience high caregiver burden.

## Method

### Study Setting and Participants

Authors M.M. and S.W. conducted an online survey to investigate the needs of carers and their experiences of providing support, which was advertised through SANE Australia, a national mental health charity. The survey targeted Australian adults (18 years or older) who currently provide postsuicide-attempt care or who have done so in the past 10 years. The project received ethics approval via the University of New England Human Research Ethics Committee (HE17-210).

A total of 834 people responded to the online survey. Reponses were excluded if the participant did not provide consent (*n* = 5), was under 18 years of age (*n* = 14), resided outside Australia (*n* = 15), or indicated that they did not know a person who had attempted suicide or did not respond to this question (*n* = 42). As the focus of this study was on those currently providing care, a further 92 were excluded where the person who attempted suicide had subsequently died. This resulted in a sample of 666 participants providing data on demographic variables. Participants' age ranged from 19 to 101 years (*M* = 46.84, *SD* = 12.93, 87.4% women). See Table E1 in Electronic Supplementary Material 1 (ESM 1) for participant characteristics. We found further 35% missing data on key study variables that resulted in a final sample of 435. The average age of participants in the final sample was 47.91 years (age range = 19–85, *SD =* 12.03, 89.7% women, 8.7% men, 1.6% reported as other).

### Measures

The following measures were used. Cronbach's α values obtained in the current study are presented in Table 1.

**Table 1:** Pearson's *r*, *M*, *SD*, and Cronbach's α values of key study variables

Variables	1	2	3	4	5	6	7	8	9	10	11	12	13	14	15
1. Closeness^a^	—	.51***	.51***	.37***	.02	−.07	−.20***	.01	−.18***	−.06	−.05	.07	−.02	.10*	.36***
2. Impact^a^		—	.40***	.33***	−.06	−.14**	−.24***	−.13*	−.31***	−.04	.05	.02	.08	.33***	.50***
3. Frequency of contact 6 months prior to attempt^a^			—	.48***	.02	−.12*	−.14*	−.03	−.18*	−.03	<.01	−.02	−.08	.06	.37***
4. Frequency of contact immediately following attempt^a^				—	.04	−.12*	−.01	−.01	−.09	−.02	.04	.01	−.03	.04	.29***
5. Confidence talking to the person about suicide attempt^a^					—	.53***	.34***	.11*	.21***	−.09	−.03	.09	−.07	−.14***	−.21***
6. Confidence supporting the person after suicide attempt^a^						—	.37***	.15**	.39***	−.03	−.06	.07	−.05	−.16**	−.36***
7. Confidence talking to others about the person's suicide attempt^a^							—	.08	.32***	<.01	−.10*	.06	−.08	−.16**	−.24***
8. Adequacy of healthcare the person received^a^								—	.34***	.06	−.02	−.06	−.15**	−.18***	−.26***
9. Adequacy of support carer received^a^									—	.01	−.04	−.01	−.11*	−.28***	−.48***
10. SOSS stigma^b^										—	−.08	−.15*	−.05	−.01	.04
11. SOSS isolation^b^											—	.07	.15***	.22***	.14**
12. SOSS glorification^b^												—	.08	.04	−.04
13. Suicidal ideation													—	.53***	.10*
14. Psychological distress														—	.44***
15. Caregiver burden															—
*M* (*SD*)	4.36 (1.04)	4.03 (1.04)	5.14 (1.27)	5.56 (.97)	3.69 (1.30)	3.57 (1.27)	3.06 (1.36)	2.85 (1.38)	2.34 (1.23)	1.30 (.59)	4.15 (.80)	2.62 (.95)	7.01 (10.42)	23.32 (9.23)	47.21 (17.32)
Cronbach's α	—	—	—	—	—	—	—	—	—	.93	.88	.88	.88	.94	.92
^a^*Note*. 1-item measure, therefore no Cronbach's α was computed. ^b^ Stigma of Suicide Scale (SOSS) = stigmatizing suicide attitudes related to stigma, isolation, and glorification, psychological distress, and caregiver burden – all measured on a 5-point Likert scale. Frequency of contact: 6 months prior to attempt and immediately following attempt, and reported adequacy of healthcare and support received measured on a 6-point Likert scale. Suicidal ideation measured on an 11-point Likert scale. *p < .05. **p < .01. ***p ≤ .001.															

#### Suicide Exposure Variables

We adapted one-item impact and closeness scales related to suicide death exposure (Cerel et al., 2015). Both closeness with the supported person (1 = *not close* to 5 = *very close*) and impact of the suicide attempt on the carer (1 = *had little effect on me* to 5 = *had a significant/devastating effect on me*) were assessed.

#### Caring Behaviors

We assessed three types of caring behaviors:

1. Frequency of contact: Frequency of contact with the person 6 months prior to the attempt and frequency of contact with the person following the attempt (1 = *infrequently* to 6 = *daily*) using one item, respectively.

2. Confidence: Three items assessed confidence: discussing suicide attempt with the person, providing support to the person after suicide attempt, and talking to others after suicide attempt (1 = *not confident* to 5 = *very confident*).

3. Reported adequacy of healthcare and support received: Carers' perception of the adequacy of healthcare the supported person received (1 = *poor* to 5 = *excellent*). We also assessed the perceived adequacy of support received by the carer (1 = *not at all supported* to 5 = *very supported*).

#### Psychological Variables

##### Stigma of Suicide Scale

The 16-item Stigma of Suicide Scale (SOSS; Batterham et al., 2013) assesses stigmatizing attitudes of community members toward suicide. It comprises three subscales (Stigma: eight items; Isolation/Depression: four items; Glorification/Normalization: four items) assessed on a 5-point Likert scale (1 = *strongly disagree* to 5 = *strongly agree*). Items are averaged, with higher scores indicating higher levels of stigma toward people who die by suicide.

##### Suicidal Ideation Attributes Scale

The five-item Suicidal Ideation Attributes Scale (Van Spijker et al., 2014) measures severity of suicidal thoughts. Items assess specific attributes of suicidal thoughts (e.g., frequency, controllability, level of distress associated with the thoughts) on a 11-point Likert scale (0 = *never/not close at all/not at all* to 10 = *always/full control/made an attempt/extremely*). Items are summed, with higher scores indicating more severe suicidal thoughts.

##### Kessler-10

The Kessler-10 (K10; Kessler et al., 2002) assesses psychological distress by asking participants to identify how often they experienced the problem (i.e., tiredness, nervousness, and hopelessness) in the last 30 days. Items, assessed on 5-point Likert scale (1 = *none of the time* to 5 = *all of the time*), are summed with higher scores indicating greater levels of distress. Scores on the K10 range from 10 to 50. The ABS (2012) categories provide a population level comparison group: 10–15 = *low levels of distress*; 16–21 = *moderate levels of distress*; 22–29 = *high levels of distress*; and 30–50 = *very high levels of distress*.

##### Caregiver Burden Scale

The 22-item Zarit Burden Interview (Zarit et al., 1980) assesses the experience of burden for those who are providing care to another. The first 21 items assess frequency on a 5-point Likert scale (0 = *never* to 4 = *nearly always*); whereas the final item assesses intensity on a 5-point Likert scale (0 = *not at all* to 4 = *extremely*). Items are summed, with higher scores indicating more burden. Scores range from 0 to 88; however, one item (from the set of the first 21 items) was inadvertently left out in the current study resulting in the total 21 items, thus the summed scores in our study ranged from 0 to 84.

### Data Analysis

Bivariate correlations (Pearson's *r*) were used to examine intercorrelations among key study variables. Hierarchical multiple regression analysis was conducted to investigate whether suicide exposure, caring behaviors, and psychological variables as a set of predictors (significant at bivariate level) would be significantly associated with caregiver burden. For supplementary analyses, we conducted: (a) moderation analysis using Hayes' PROCESS (v3.5; 2017) macro testing the moderated effect of psychological distress on the relationship between suicidal ideation and caregiver burden; and (b) independent-samples *t* tests to investigate differences between carers who reported to be “supported” versus “not well supported” on key psychological factors.

## Results

### Descriptive Statistics

Table 1 provides intercorrelations and descriptive statistics for the key study variables (see Table E2 in ESM 1 for interpretation of the sample mean scores).

As expected, participants reported high levels of relational closeness with the supported person, impact of the suicide attempt, frequency of contact, stigma related to isolation, suicidal ideation, and psychological distress were associated with high caregiver burden. Respondents who reported confidence discussing the suicide attempt, providing support, talking to others after suicide attempt, adequacy of healthcare the supported person received, and support they received experienced less caregiver burden.

We computed correlations of three characteristics (gender: male/female; location: metro/non-metro; and time since last attempt), two of which (gender and location) have been shown to be related with caregiver burden (Ehrlich et al., 2015). We found being female, *r*(426) = .24, *p* < .001 and time since last attempt, *r*(433) = −.11, *p* = .026, were significantly correlated with caregiver burden, and were used as covariates in the multivariate model. Geographic location was not significantly associated with caregiver burden, *r*(433) = −.01, *p* = .892.

### Hierarchical Multiple Regression Analysis

All relevant test assumptions were checked. The results, summarized in Table 2, revealed that the overall model was significant, *F*(14, 413) = 31.50, *p* < .001, that is, as a set all predictors (including covariates) explained 52% of variance (*R*^2^) in caregiver burden. Covariates – Step 1: Δ*F*(2, 425) = 16.42, *p* < .001, *R* = .27, Δ*R*^2^ = .07; suicide exposure-related closeness and impact – Step 2: Δ*F*(2, 423) = 66.38, *p* < .001, *R* = .54, Δ*R*^2^ = .22; caring behaviors (*frequency of contact*: 6 months prior to attempt and immediately following attempt; *confidence* in discussing suicide attempt with the person, confidence in providing support to that person, and talking to others about suicide attempt; and *reported adequacy of healthcare* the person received and *adequacy of support* the carer received) – Step 3: Δ*F*(7, 416) = 17.21, *p* < .001, *R* = .67, Δ*R* ^2^ = .16; and psychological variables (SOSS isolation, suicidal ideation, and psychological distress) – Step 4: Δ*F*(3, 413) = 18.29, *p* < .001, *R* = .72, Δ*R*^2^ = .06 – explained significant amounts of variance in caregiver burden, respectively. Specifically, being female (β = .11, *p* < .05, *sr*^2^ = 1%), reported closeness with the supported person (β = .11, *p* < .05, *sr*^2^ = 1%), high impact of the suicide attempt (β = .17, *p* < .001, *sr*^2^ = 2%), high frequency of contact 6 months prior to attempt (β = .12, *p* < .01, 1%), and high psychological distress (β = .32, *p* < .001, *sr*^2^ = 5%) contributed significantly to high caregiver burden. On the other hand, carers who reported having confidence in supporting the person (β = −.14, *p* < .01, *sr*^2^ = 1%), adequate level of healthcare the person received (β = −.09, *p* < .05, *sr*^2^ = 1%), adequate support the carer received (β = −.20, *p* < .001, *sr*^2^ = 3%), and high suicidal ideation (β = −.11, *p* < .05, *sr*^2^ = 1%) experienced significantly less caregiver burden.

**Table 2:** Summary of hierarchical multiple regression analysis: suicide exposure, caring behaviors, and psychological variables as predictors of caregiver burden in a sample of carers

Predictors	*R*	*Adj R* ^*2*^	*B*	95% CI for *B*		β	*sr* ^*2*^
				LL	UL		
Full model	.72***	.50					
Covariates^a^							
Gender			6.53	2.33	10.73	.11*	.01
Time since last attempt			−0.12	−.90	.65	−.01	<.01
Exposure to suicide							
Closeness			1.75	.30	3.20	.11*	.01
Impact			2.84	1.39	4.28	.17***	.02
Caring behaviors							
Frequency of contact (6 mo prior to attempt)			1.69	.52	2.86	.12**	.01
Frequency of contact (immediately following attempt)			1.16	−.26	2.59	.07	<.01
Confidence talking to the person about suicide attempt			−0.76	−1.87	.34	−.06	<.01
Confidence supporting the person after suicide attempt			−1.88	−3.00	−.64	−.14**	.01
Confidence talking to others about the person's suicide attempt			0.45	−.54	1.43	.04	<.01
Adequacy of healthcare the person received after attempt			−1.10	−2.01	−.20	−.09*	.01
Adequacy of support the carer received			−2.76	−3.90	−1.61	−.20***	.03
Psychological variables							
Attitudes towards suicide: Isolation			1.24	−.28	2.75	.06	<.01
Suicidal ideation			−0.18	−.32	−.04	−.11*	.01
Psychological distress			0.59	.42	.76	.32***	.05
*Note. R* = multiple correlation between the observed and predicted values of the DV, *Adj R*^2^ = adjusted amount of variation in the outcome variable that is accounted for by the model, *B* = unstandardized beta coefficients, CI = confidence intervals, LL = lower limit, UL = upper limit, β = standardized beta coefficients, and *sr*^*2*^ = squared semipartial correlation (amount of unique variance in the dependent variable explained by a predictor after controlling for the other predictors in the model). Results reported in the table correspond to Step 4 in the model. ^a^Personal characteristics: Gender: 1 = male; 2 = female; Time since last attempt: 1 = less than 1 month; 2 = 1–6 months; 3 = 7–12 months; 4 = 1–2 years; 5 = 3–5 years; 6 = 6–10 years. *p < .05. **p < .01. ***p < .001. *N* = 428 for this analysis (seven cases reporting their gender as “other” were excluded). Bold values indicate statistical significant effects.							

After controlling for other predictors in the model, time since last attempt, frequency of contact immediately following attempt, confidence talking to the person about their suicide attempt, confidence talking to others about the person's suicide attempt, and SOSS isolation did not explain significant unique variance in caregiver burden (all values *p* > .05).

**Figure 1:**
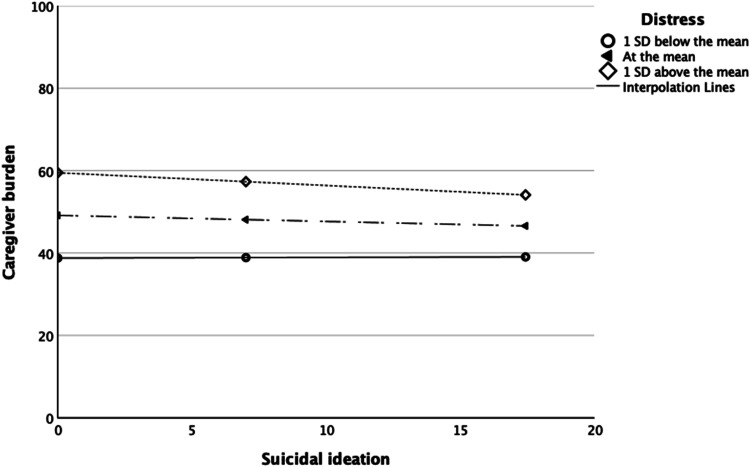
Psychological distress as a moderator of the relationship between suicidal ideation and caregiver burden.

Contrary to the finding of a significant positive bivariate correlation between suicidal ideation and caregiver burden, this relationship became negative in the multivariate model (β = −.11, *t* = −2.60, *SE* = 0.07, *p* < .01), after controlling for other predictors. To further understand this, we conducted a moderation analysis to examine whether psychological distress moderated the relationship between suicidal ideation and caregiver burden. Results, using 5,000 bootstrapped samples and estimates, suggested an overall significant model, *F*(3, 431) = 42.08, *p* < .001, *R* = .48, *R*^2^ = .23. The main effect of suicidal ideation on caregiver burden was not significant, β = .26, *t* = 1.02, *SE* = 0.26, *p* = .308, 95% CI [−.24_LB_, .77_UB_]. However, we found the main effect of psychological distress (β = 1.12, *t* = 10.47, *SE* = 0.11, *p* < .001, 95% CI [.91_LB_, 1.33_UB_]), and the interaction effect (suicidal ideation × distress: β = −.02, *t* = −2.28, *SE* = 0.01, *p* = .023; 95% CI [−.03_LB_, −.002_UB_]; see Figure 1) significant. Simple slope analyses showed that low levels of distress (1 *SD* below the mean; β = .02, *t* = .10, *SE* = 0.16, *p* = .923, 95% CI [−.30_LB_, .33_UB_]) and average (at the mean) levels of distress (β = −.15, *t* = −1.40, *SE* = 0.11, *p* = .162, 95% CI [−.35_LB_, .06_UB_]) did not significantly moderate the relationship between suicidal ideation and caregiver burden. However, high levels (1 *SD* above the mean) of distress showed a significant moderated effect (β = −.31, *t* = −3.72, *SE* = 0.08, *p* < .001, 95% CI [−.47_LB_, −.15_UB_]). That is, at high levels of distress, as suicidal ideation scores increased, participants reported less caregiver burden.

We speculated this reduction in caregiver burden among distressed carers with suicidal ideation may be a result of their contact with support services. That is, high distress with high suicidal ideation might prompt carers to *seek* support for themselves having been involved in support for the person and thus more aware of support available to them, thus reducing perceptions of caregiver burden. To understand this, we examined participants' distress and suicidal ideation as a function of receiving adequate support as a carer. We categorized participants into two groups: (1) those who reported being “not well supported” (54.5%) based on their responses as 1 = *not at all adequately supported* or 2 = *supported a little* on a 1-item scale assessing feeling supported to care for the person; and (2) those who reported being “supported” (18.9%) based on their responses as 4 = *adequately supported* or 5 = *very adequately supported*. We did not include participants who responded 3 = *neutral* in this analysis. Note: the definition of “support” was open to participant interpretation. Independent-samples *t* test results showed that participants in the “not well supported” group reported higher scores on suicidal ideation, psychological distress, and caregiver burden compared to the participants in the “supported” group (see Table E3 in ESM 1). That is, carers reported significant reductions in psychological distress and suicidal ideation, in addition to caregiver burden, after having received adequate “support” compared with those who reported being not well supported.

## Discussion

Carers of people who have made a suicide attempt are a highly distressed group and are at heightened risk of suicide themselves (O'Dwyer et al., 2013). However, little is known about the additive effects of suicide exposure, caring behaviors, and psychological variables on caregiver burden. Understanding the risk and protective factors would help inform strategies on how to best support carers of individuals who have attempted suicide in the development of future interventions and support services for carers.

This study identified that being female, feeling close to the supported person, high impact of suicide attempt, high frequency of contact pre-attempt, and high psychological distress were significant positive contributors to the caregiver burden. These findings are consistent with previous research on carers of people with mental illness (Hielscher et al., 2019) that the impact of caregiving, feeling close to the person, and a perceived inability to avert the attempt given a high frequency of contact with the person led to the experience of high caregiver burden. Combining findings with previous literature highlights that females are more likely to take on caring roles compared with males (Diminic et al., 2019), where a doubling or more of working hours impacts on the mental health of female carers (Treichel et al., 2020). This may be in conjunction with less choice about the role, making the caregiver experience more impactful (O'Connor & Forgan, 2007). Specifically in relation to caring for a person after a suicide attempt, our findings indicated that a carer's confidence in providing support, adequate healthcare the supported person received, and adequate support the carer received resulted in experiencing less caregiver burden. Contrary to our prediction, holding stigmatizing views of suicide and confidence talking to the person and others about the attempt were not significantly related to caregiver burden. This adds nuance to prior reports that carers may feel unable to disclose the suicide attempt due to fear of stigma and shame (McLaughlin et al., 2016; Spillane, 2019).

A counterintuitive finding related to those participants with heightened suicidal ideation who also reported less caregiver burden in the multivariate model. Moderation analysis found this negative relationship between suicidal ideation and caregiver burden was only evident for those carers who reported high psychological distress. We speculate that this may be a result of existing knowledge of, and access to, support services due to their caregiving role which, in turn, is associated with less caregiver burden. That is, high psychological distress prompts carers to seek their own support. Our post hoc analysis suggested that through receiving support, carers also reported significant reductions in suicidal ideation and psychological distress in addition to lower caregiver burden than that of those who reported feeling unsupported. Our findings therefore have implications for the development of specific – and adequate – supportive interventions for carers with or without a bereavement response to reduce their suicide risk (Bhullar et al., 2021; Maple et al., 2017; Pitman et al., 2014). To our knowledge, this is the first study to examine the combined contributions of suicide exposure, caring behaviors, and psychological variables in caregiver burden. Further research is needed with more sensitive tools that can examine what functions of support adequacy, timeliness, and activities are important to meet the needs of the carers. Further research could also investigate the longitudinal trajectories of risk and protective factors implicated in caregiver burden.

## Limitations

This study used a cross-sectional, online survey of self-identified carers and is not representative of the carer population. The data were collected at one point in time, thus limiting our ability to determine the longitudinal outcomes for carers as well as understanding of the differential effects of “seeking support” and “receiving adequate support” on distress and suicidal ideation. Future research could employ a longitudinal design to tease apart the temporality of how seeking support and receiving adequate support for people caring for those with complex challenges affects caregiver burden over time. Our sample is skewed, with by far the majority of our sample being female. However, a relatively large sample size enabled us to detect a large effect size for our main findings.

## Conclusion

Those providing informal care to a person who has attempted suicide play an important role in suicide prevention. Yet, this activity can result in suicide risk and other adverse outcomes for the carer. Taking care of the carers most at risk of suicide and self-harm is a cost-effective and meaningful way to reduce distress in this highly distressed group. Providing targeted support to carers when they are already in contact with services, via their caregiving role, is a modifiable factor to enhance the psychological wellbeing of the carers of suicide attempt survivors.

## Electronic Supplementary Material

The electronic supplementary material is available with the online version of the article at https://doi.org/10.1027/0227-5910/a000836

**ESM 1.** Details of participant characteristics and interpretation of sample mean scores on key variables
