# Supplementary material for: Predictors of Caregiver Burden Among Carers of Suicide Attempt Survivors
Source: Crisis. 2021 Dec 17;44(1):41–8. doi: 10.1027/0227-5910/a000836 (PMC9909369; doi:10.1027/0227-5910/a000836)
Supplement: Supplementary file 1 [file cri_44_1_41_esm1.pdf]

https://doi.org/10.1027/0227-5910/a000836

Electronic Supplementary Material 1

Table E1: Participant Characteristic

Variables	*N*	%
Gender		
Male	68	10.2
Female	582	87.4
Transgender	4	0.6
Other	12	1.8
Aboriginal and/or Torres Strait Islander descent		
Yes	15	2.3
No	651	97.7
Geographic location		
Metropolitan	348	52.3
Regional	216	32.4
Rural	94	14.1
Remote	8	1.2
Number of people known who have attempted suicide		
1	174	26.1
2-3	291	43.7
4-5	98	14.7
5-10	49	7.4
10+	53	8.0
Missing	1	0.2
Relationship to the person who attempted suicide		
Parent	61	9.2
Family relation (cousin, aunt, grandparent)	42	6.3
Child	168	25.2
Friend	156	23.4
Sibling	39	5.9
Colleague	8	1.2
Partner	71	10.7
Other	58	8.7
Missing	63	9.5
Number of known suicide attempts by the person who attempted suicide		
1	189	28.4
2-4	317	47.6
5-10	68	10.2
More than 10	29	4.4
Missing	63	9.5
Time since last attempt by the person who attempted suicide		
Less than 1 month	59	8.9
1-6 months	131	19.7
7-12 months	73	11.0
1-2 years	124	18.6
3-5 years	103	15.5
6-10 years	113	17.0
Missing	63	9.5

https://doi.org/10.1027/0227-5910/a000836

Table E2: Summary of Sample Mean Scores of Key Study Variables

**Variables**	**Interpretation of Sample Mean Scores**
Closeness	High levels of closeness with the person who attempted suicide; the mean score was above 4 on the 5-point Likert scale
Impact	High levels of perceived impact; the mean score was above 4 on the 5-point scale
Frequency of contact 6 months prior to attempt	High levels of frequency of contact during the six months prior to attempt; the mean score was over 5 on the 6-point scale
Frequency of contact immediately following attempt	High levels of frequency of contact immediately following attempt; the mean score was over 5 on the 6-point frequency scale
Confidence talking to the person about suicide attempt	Moderate levels of confidence talking to the person about their suicide attempt; the mean score fell above the mid-point on the 5-point scale
Confidence supporting the person after suicide attempt	Moderate levels of confidence supporting the person after their suicide attempt; the mean score fell above the mid-point on the 5-point scale
Confidence talking to others about the person's suicide attempt	Moderate levels of confidence talking to others about the person's suicide attempt; the mean score was just over the mid-point on the 5-point scale
Adequacy of healthcare the person received	Low levels of reported adequacy of healthcare the person received; the mean score was below 3, the mid-point on the 5-point scale
Adequacy of support carer received	Low levels of reported adequacy of support carer received; the mean score was below 3, the mid-point on the 5-point scale
SOSS stigma	Low levels of suicide attitudes related to stigma; the mean score was well below the mid-point on the 5-point scale
SOSS isolation	High levels of stigmatising attitudes related to isolation; the mean score was above 4 on the 5-point scale
SOSS glorification	Low levels of stigmatising attitudes related to glorification; the mean score was well below the mid-point on the 5-point scale
Suicidal ideation	High levels of suicidal ideation; the mean score was just over 7, well above the mid-point on the 11-point scale
Psychological distress	High levels of average psychological distress levels.<br>Distress levels (ABS, 2012): Very high (24.4%), High (26.4%), Moderate (25.7%), and Low (23.4%)
Caregiver burden	High levels of caregiver burden; the mean score was above the mid-point.

https://doi.org/10.1027/0227-5910/a000836

Table E3: Summary of Group Differences on Key Psychological Variables

Variables	Not well supported Group (*n*=237)	Supported Group (*n*=82)	*t-values* (317)	Cohen's *d*
Suicidal ideation	7.52 (10.74)	4.88 (7.85)	2.05*	.28
Psychological distress	25.04 (8.84)	19.44 (8·77)	4.96***	.64
Caregiver burden	54.17 (13.53)	34.54 (19.51)	10.03***	1.17

Note. $^{*}p < .05$, $^{***}p < .001$. Cohen's *d effect size guidelines*: .20 (small); .50 (medium); and .80 (large).
